# Glances at the Hospitals

**Published:** 1900-12-01

**Authors:** 


### GLANCES AT THE HOSPITALS.

### THE ROYAL BERKSHIRE HOSPITAL.

It would seem from the treasurer's report that the total ordinary receipts amounted to £7,073, the total ordinary disbursements to £8,937, and that at the end of the year there was due to the treasurer a sum of £3,355. Special efforts were made during the year to improve the financial position of the hospital, and these efforts resulted in an addition of £11,831 to the invested funds, and about £700 to the subscription list. But there has also been increase in the expenditure, partly caused by an increase in the number of patients, and it is anticipated that the income for 1900 will "be insufficient by a considerable sum to meet our ordinary expenditure." Looking at the results of former years there can hardly be a doubt that there will be a deficit; and, if annual subscriptions are not forthcoming, surely the committee should take some step to reduce the expenses. The average duration of residence in the hospital was 29 days. A statement showing the cost per bed occupied, and the average weekly cost per patient, would be desirable. The house surgeon's report states that 136 patients were in the hospital on January 1st, and that 1,419 were admitted during the year, making a total of 1,555. Of these 672 were discharged cured, 399 relieved, 158 were made out-patients, 56 not benefited, 12 for other reasons, 130 died, and 128 remained under treatment on December 31st, 1899. As to the out-patients, 547 remained from 1898, 2,127 were admitted 2,203 were discharged, and 471 remained. In addition to these there were 9,544 minor accidents and casualties. The in-patients' diseases are classified on pages 43 and 44, and the number of patients suffering from each disease is given. Including 212 minor operations, there were 1,020 operations performed during the year. The anæsthetic used is not stated.

